# How the Porous Transport Layer Interface Affects Catalyst Utilization and Performance in Polymer Electrolyte Water Electrolysis

**DOI:** 10.1021/acsami.3c04151

**Published:** 2023-07-17

**Authors:** Carl Cesar Weber, Jacob A. Wrubel, Lorenz Gubler, Guido Bender, Salvatore De Angelis, Felix N. Büchi

**Affiliations:** † Electrochemistry Laboratory, Paul Scherrer Institut, 5232 Villigen PSI, Switzerland; ‡ National Renewable Energy Laboratory, Golden, Colorado 80401, United States

**Keywords:** hydrogen, PEM electrolysis, polymer electrolyte water electrolysis, active catalyst layer, porous transport layer, interface PTL/CL, catalyst utilization, iridium loading

## Abstract

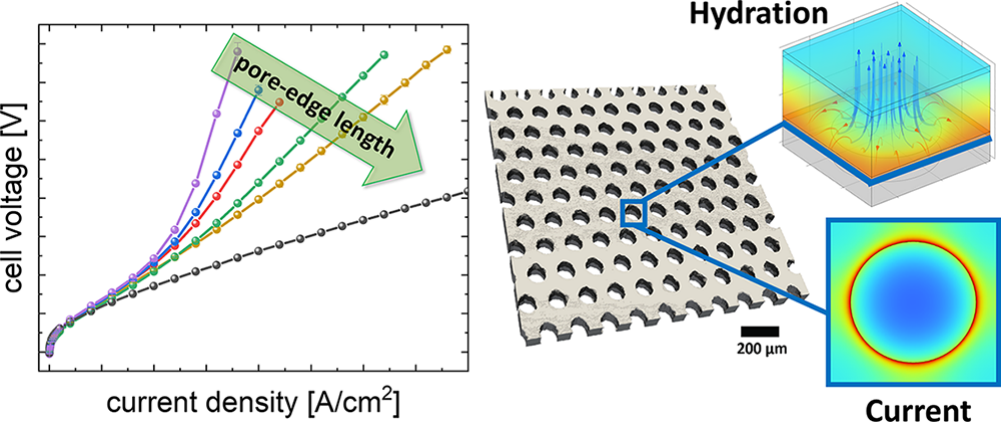

Cost reduction and fast scale-up of electrolyzer technologies are essential for decarbonizing several crucial branches of industry. For polymer electrolyte water electrolysis, this requires a dramatic reduction of the expensive and scarce iridium-based catalyst, making its efficient utilization a key factor. The interfacial properties between the porous transport layer (PTL) and the catalyst layer (CL) are crucial for optimal catalyst utilization. Therefore, it is essential to understand the relationship between this interface and electrochemical performance. In this study, we fabricated a matrix of two-dimensional interface layers with a well-known model structure, integrating them as an additional layer between the PTL and the CL. By characterizing the performance and conducting an in-depth analysis of the overpotentials, we were able to estimate the catalyst utilization at different current densities, correlating them to the geometric properties of the model PTLs. We found that large areas of the CL become inactive at increasing current density either due to dry-out, oxygen saturation (under the PTL), or the high resistance of the CL away from the pore edges. We experimentally estimated the water penetration in the CL under the PTL to be ≈20 μm. Experimental results were corroborated using a 3D-multiphysics model to calculate the current distribution in the CL and estimate the impact of membrane dry-out. Finally, we observed a strong pressure dependency on performance and high-frequency resistance, which indicates that with the employed model PTLs, a significant gas phase accumulates in the CL under the lands, hindering the distribution of liquid water. The findings of this work can be extrapolated to improve and engineer PTLs with advanced interface properties, helping to reach the required target goals in cost and iridium loadings.

## 1 Introduction

The quest for suitable mid- and long-term energy storage solutions necessary to cope with the fluctuating nature of most renewable electricity sources is developing at a tremendous pace. Hydrogen produced by water electrolysis is a promising energy vector due to its applicability not only for mobility or grid energy storage but also in various other industries (e.g., fertilizers, steel, and chemical refinement). Among the available electrolysis technologies, polymer electrolyte water electrolysis (PEWE) is a promising technology that is well-suited to be coupled with fluctuating renewable power sources.^1^ However, high capital and operational expenditures (CAPEX and OPEX) are still hindering its commercial breakthrough.^2^ The high price and the limited supply of iridium, which is required for catalyzing the oxygen evolution reaction, are most likely the main limiting factors.^3^ While a significant amount of research has focused on trying to replace iridium as a catalyst,^4^ the applicability of non-Ir-based materials at an industrial scale will still require a long time to implement. Therefore, there is an urgent need to reduce the required amount of noble metals in the catalyst layers (CL), minimizing the iridium-specific power density to targets from currently about 0.65–0.75 to 0.01–0.05 g_Ir_/kW.^3,5^

Reducing catalyst loadings without altering the catalyst’s composition and intrinsic activity has the direct consequence of reducing the CL thickness.^6^ In this scenario, it is essential to utilize the available catalyst as efficiently as possible. Recent studies have shown that the structure of the PTL can have an essential impact on CL utilization.^7−12^ Several studies have investigated the bulk properties of the PTL^13−17^ and the respective fluidic transport during operation.^18−28^ A significant influence affecting CL utilization is related to the surface properties of the PTL rather than the bulk features.^7,8,10−12^ This is linked to two main reasons: (i) the poor electronic in-plane (IP) conductivity of typical CLs, caused by the disruption of the electronic percolation network of the CL structure and (ii) the in-plane mass transport limitation in the porous structure of the CL under the contact points between PTL and CL.

Schuler et al. investigated the PTL morphology and surface properties of different fiber-based PTLs and correlated them to their respective electrochemical performance.^7,8^ The authors found that a rough PTL surface structure can lead to mechanical deformation and cracks in the CL, which leads to losses in CL utilization and has a strong impact on all overpotentials. Also, Lopata et al. studied the PTL/CL interfaces showing that PTL properties, such as the average pore and grain diameter, impact cell performance with a stronger effect when reducing the catalyst loadings.^9^ Several other studies have tried to improve the PTL/CL interface using different approaches including laser structuring,^29^ the introduction of a microporous layer,^30,31^ nanofiber interlayers,^32^ and others.^33−36^ However, a comprehensive understanding of how this interface affects CL utilization is still elusive.

Model PTLs with very well-defined features are a useful tool to study limiting effects occurring in the cell during operation. Several publications have studied PTLs with well-defined features. Mo et al.^37,38^ and Kang et al.,^39−41^ reported the use of thin and tunable Ti-PTLs fabricated via lithography and chemical etching, with the possibility to produce pores of different sizes. By using a transparent cell and optical imaging, the authors showed that oxygen bubbles seem to form mostly at the edge of the pores, indicating that the electrochemical activity could be concentrated at this location. While this seems to be a reasonable deduction due to the poor electronic IP conductivity of typical PEWE CLs, the authors did not discuss the effect of mass transport under the PTL lands. It is known from the literature,^42−45^ that the CL has porosities in the range of 15–70% depending on the ionomer content, and therefore IP mass transport can occur even without direct contact with the open pore space of the PTL.

Kim et al. used titanium mesh PTLs with various pore openings to tailor the CL interface where reducing the mesh opening size resulted in lower ohmic losses.^46^ Kang et al. used thin 2D-PTLs^40^ with different pore sizes and porosities showing that both can have an impact on performance but with a higher contribution given to porosity. The authors found that small pore sizes and high porosity lead to better performance, and the activity seems to be concentrated only around the pore edges.

Although the implementation of such materials in large-scale electrolyzers is probably not feasible due to the complex and expensive manufacturing process, the information that we can obtain to understand the impact of the PTL/CL interface on PEWE performance can be very useful. One of the main advantages of having well-defined, controllable, and simple features is that the measured overpotentials can be correlated more easily to the geometric features of the PTL/CL interface. In a modeling study, Kang^47^ found that the IP current drop across the 2D-PTLs pore is considerable (down to less than 1% of the current at the edge) and highly dependent on the CL IP conductivity. However, the role of the CL below the PTL land was not reported. Wrubel et al.^48^ employed a more extensive multiphysics model coupling electrochemistry and multiphase transport in an electrolyzer cell with thin the 2D-PTL. The authors studied the effect on alignment with the flow field and respective land blockage. In this work, it was shown that large pores that are closely spaced (i.e., higher porosity) perform better because they can prevent oxygen buildup in the CL. Furthermore, it was shown that the anisotropic periodic structure of the 2D-PTLs can lead to a non-uniform water distribution in the membrane with the consequence of localized dry-out, which in turn increases ohmic losses in the cell.

In this work, we use well-defined 2D-PTLs model materials to understand how pore size, pore spacing (particle/fiber size), and porosity of the PTL affect CL utilization and the overpotentials in PEWE cells. Characterization of the electrochemical performance and an in-depth overpotential analysis are used to correlate interface geometry with CL utilization. Furthermore, the model of Wrubel et al.^48^ was adapted to a 3D multiphysics model, to support our hypothesis based on experimental data on membrane dry-out and the current density distribution in the CL under the PTL land. The findings in this work can be extrapolated to commonly used porous materials, understanding how the surface properties of real-world PTLs (Ti-fiber felts, Ti-particle sintered materials) influence catalyst utilization, ultimately leading to the engineering of PTL materials with improved interface properties to achieve and increase PEWE performance.

## 2 Results and Discussion

### 2.1 2D-Porous Transport Layers: Structure Description and Initial Tests

**Figure 1:**
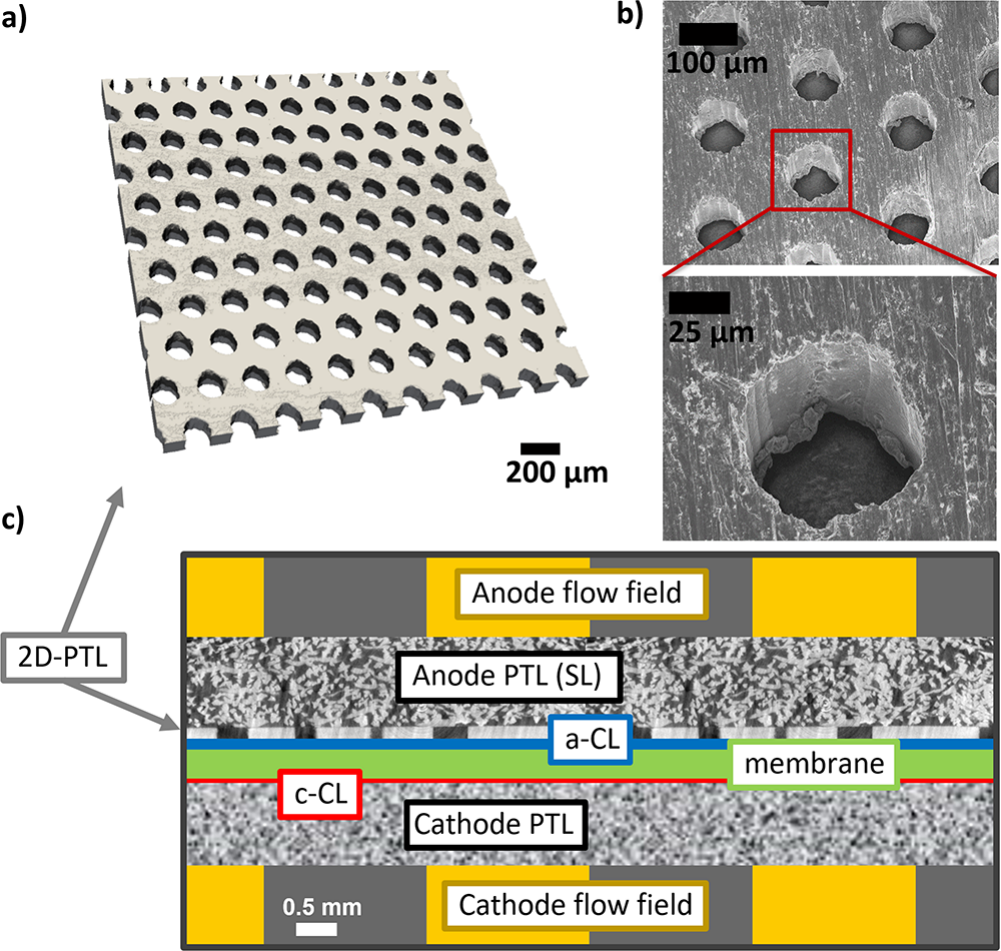
(a) 3D surface renderings from XTM data of a selected 2D-PTL with 100 μm pore size and 26% porosity, (b) SEM images of the laser-drilled pores, (c) schematic illustration of a cross-section of typical cell configuration with the 2D-PTL at the interface between PTL (SL) and CL (sketched CCM not on scale).

A matrix of five 2D-PTLs with three different pore sizes (each with equal porosity of 26% but with varying pore diameters of 100, 200, and 400 μm) and three different porosities (at constant pore size of 200 μm and ∼9, 26, and 43% porosity) were designed. While the lower porosities (i.e., 9%) are used for investigative purposes and the higher porosities are in the range of commercial PTLs (e.g., 50% for Ti-fiber felts and 30–40% for Ti-particle sintered materials). Higher porosities (>50%) are difficult and costly to achieve by laser drilling due to the high pore density that is needed. All 2D PTLs were 140 μm thick. Table 1 lists the designed PTLs and their respective features. In Figure 1a, a surface rendering from X-ray tomographic microscopy (XTM) data of a selected 2D-PTL is presented with the specific pore arrangement, and Figure 1b shows a representative SEM of the sample with 100 μm and 26% porosity. The relationship between the pore diameter, inter-pore distance *L,* and the porosity is given by the following formula, similar to the one given by Kang et al.:^40^
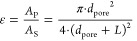
1

**Table 1:** Characteristics of PTLs Used in this Study: Pore Diameter, Porosity, IP Spacing or Fiber Diameters, Specific Pore Edge Length (SPEL), and Nominal PTL Thickness

PTL type	pore diameter [μm]	porosity [%]	*x*/*y*-spacing or fiber diameter [μm]	specific pore edge length [cm/cm^2^]	nominal PTL thickness [mm]
100 μm 26%	100	26	74	104	0.14
200 μm 26%	200	26	148	52	0.14
400 μm 26%	400	26	295	26	0.14
200 μm 43%	200	43	69	87	0.14
200 μm 9%	200	8.7	402	17	0.14
Ti-felt SL^7^	45.5	74	17.4		1.0
Ti-felt reference^17^	29.2	52.5	20.8		1.0

where ε represents the porosity, *A*_P_ is the area of a single pore, and *A*_S_ is the total solid area of a repeat unit. Using the repeat unit approach (similar to the packing density of crystal structures), the porosity can be tuned with the pore diameter *d*_pore_ and the pore–pore distance *L* (edge to edge). In Figure S1a, an SEM image with the respective sketched distances is shown for a selected sample with a 200 μm pore diameter and 148 μm pore–pore distance, resulting in a porosity of 26%.

All parameters for the different PTLs used in this study are summarized in Table 1. From the measured pore sizes and porosities, the specific pore edge length (SPEL) referring to the total length of the edges of the pores was calculated as follows:
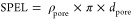
2with ρ_pore_ as the pore density (in pores per cm^2^) and *d*_pore_ as the pore diameter.

The surface roughness of the 2D-PTL, when neglecting the pore edge, is very low in comparison to standard PTL materials (such as titanium felts). However, it is important to consider that uncompressed regions of the CCM exist in the pore areas, leading to poor electrical contact and CL deformation. Therefore, we can derive three PTL/CL interfacial regions: (i) the area under the PTL land, which comprises a smooth and compressed CCM, (ii) the area in the open pore space, which is not compressed by the PTL, and (iii) the area at (and close to) the circumference of the pore (or SPEL), which is an intermediate region. In Figure S1b, an SEM image of a post-test CCM is shown with the three important interfacial regions highlighted.

For an initial test assessment, we compared the performance of using: (i) a pure 2D-PTL, (ii) a combination of a Ti-felt support layer (SL), and the 2D-PTL, and (iii) a standard Ti-felt at the anode side while leaving the cathode side constant (Ti-felt). The polarization curves and HFR measurements are shown in Figure S2. We observed an extreme increase in voltage in combination with a high increase in HFR when using the pure 2D-PTLs, especially at high current densities. This effect was much lower when using the combination of the SL and 2D-PTL. At low current densities (up to 0.4 A/cm^2^) both measurements using 2D PTL (with and without a SL) are almost identical and exhibit slightly better performance than with the Ti-felt.

The increase in HFR at higher current density is likely related to the drying of the membrane located under the land of the flow fields. These regions have no direct access to liquid water because, due to the geometry of the 2D-PTLs, there is no open porosity in the IP direction. Therefore, the membrane gets hydrated only from the pores of the CL and the water transport through the membrane itself.

While at low current density (<0.4 A/cm^2^), the humidification of the membrane via the CL porosity is sufficient; however, with increasing current densities, the regions under the land get saturated with oxygen. This leads to drying of the membrane and severe losses in proton conductivity, leading to an increase in the HFR. This effect is suppressed when the 2D-PTL is combined with a Ti-felt PTL. The relatively thick and highly porous (74%) SL ensures sufficient water distribution and gas removal in the regions under the flow fields lands, minimizing the effect of land blockage.

For this reason, for the rest of this study and comparison between the different 2D-PTLs, we used a Ti-felt as a support/diffusion layer between the 2D PTL and the flow field, which ensures equal water/gas diffusion over the entire 2D-PTL. The SL PTL was composed of a highly porous Ti-felt material from Bekaert (see Table 1). Hence, the 2D-PTL was used as an additional interfacial layer between the support layer and the CL, analogous to a microporous layer but with larger and well-defined features. A schematic illustration showing the typical cell configuration is shown in Figure 1c.

### 2.2 Performance and Overpotentials Analysis

From the large difference in pore sizes (100–400 μm) and porosities (9–43%) of the 2D-PTL, we can expect major differences in the electrochemical performance. In previous studies, Kang et al. showed that high porosity and small pore sizes show the best performance, with porosities having a higher impact than pore sizes.^40^ In this investigation, they demonstrated high performance using only ultra-thin 2D-PTLs (thickness of 25 μm). However, the problem of flow field land blockage while using 2D-PTLs is not mentioned or discussed.

#### 2.2.1 PEWE Performance Comparison and Kinetic Analysis

**Figure 2:**
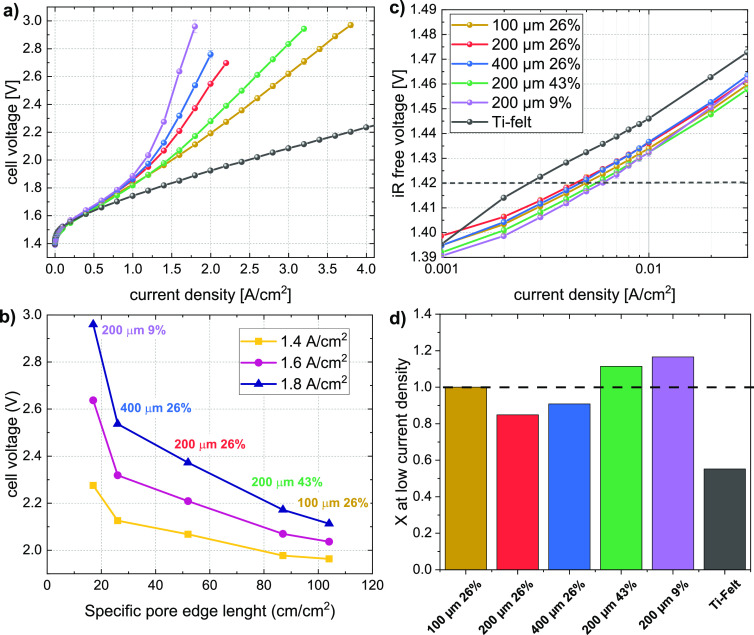
(a) Comparison of the polarization curves of the used two-dimensional PTL at 80 ° C and ambient pressure, (b) cell voltages at 1.4, 1.6, and 1.8 A/cm^2^ vs the specific pore edge length (circumference length) of the 2D-PTLs, (c) logarithmic plot of the *iR*-corrected voltages at low current densities (i.e., Tafel plots), and (d) relative CL utilization at low current density taken at 1.42 V.

Up to the interface between the support layer and the 2D-PTL, we can assume the water distribution to be very similar for all 2D-PTLs. Therefore, for each 2D-PTL, we can relate the differences in electrochemical performance to the variation of their interfacial properties. Figure 2a shows the polarization curves of all five 2D-PTLs at 80 °C and ambient pressure. For low current densities (<0.4 A/cm^2^), all PTLs lead to similar cell performances while deviations start to arise at certain critical current densities (different for each PTL). We can observe the following trends in the cell voltages: for equal porosity of 26%: 100 μm < 200 μm < 400 μm and for equal pore sizes (200 μm) 43% < 26% < 9%. These trends show that small pore sizes and high porosity is favored and yield lower cell voltages. This observation is in line with the findings of Kang et al.,^40^ who observed the same trends, but with porosity having a higher impact than the pore size. Differences might arise from different boundary conditions such as the type of CCM, the lack of a diffusion layer, or the type of cell. In our results, the best performance is observed for the sample with the smaller pore sizes and medium porosity (100 μm 26%) followed by the sample with 200 μm pore sizes and the highest porosity (50%). This hints that, in our specific case, smaller pore sizes have a greater impact on performance than higher porosity.

A further characteristic that defines the different 2D-PTLs is the SPEL (or circumference length in cm/cm^2^). In Figure 2b, the cell voltage at three different high current densities of 1.4, 1.6, and 1.8 A/cm^2^ vs the SPEL of the 2D-PTLs, is shown. We observe a clear trend of decreasing cell voltage at increasing SPEL. In previous studies by Kang et al. and Mo et al.,^37,38,40^ it was hypothesized that, in the CL, only the region close to the SPEL is active. The CL regions in the pore space were considered not to be utilized due to the low CL electronic IP conductivity. However, the activity in the regions under the PTL (which in our case corresponds to at least half of the active area) was not discussed.

The land area of the PTL has good transport of electrons due to its direct contact with the CL. However, the oxygen and water transport occurs only via the open porosity of the CL or from the water in the membrane. While this was only vaguely discussed by the authors, it can be assumed that the initial mass transport must occur through the CL itself. In a recent study, De Angelis et al. characterized the 3D structure of a typical PEWE CL via high-resolution ptychographic X-ray imaging, showing porosities that are in the range of ∼40%,^44^ therefore high enough to ensure fluidic transport.

Figure 2c shows the *iR*-corrected Tafel plots for all five PTLs. All curves are almost identical (differences are in the order of ∼5 mV), especially considering the large difference in pore size and porosity. Table 2 lists the kinetic parameters for all 2D-PTLs. Only minor discrepancies in Tafel slopes were observed, showing slightly higher Tafel slopes for the two samples with the lowest SPEL. We believe this slight discrepancy in the Tafel slopes is an artifact related to mass transport effects or access to the catalyst sites, affecting the slopes even at low current densities. In fact, we calculate Tafel slopes using points between 3 and 30 mA/cm^2^, and the slopes for 200 μm 9% and 400 μm 26% already start to deviate from their linear behavior at ∼7 mA/cm^2^. Therefore, since the same catalyst material and the CL fabrication were used (commercial CCMs from Greenerity), we can expect that the true Tafel slope is equal for all 2D-PTLs.

**Table 2:** Estimated Tafel Slopes (3–30 mA/cm^2^), Activity at a Given iR-Free Voltage @ 1.42 V, and the Respective Relative CL Utilization Factor *X*_kin_ with the 100 μm 26% Sample as the Reference

PTL type	Tafel slope [mV/dec]	*j*_@1.42V_ [mA/cm^2^]	CL utilization *X*_kin_
100 μm 26%	49.1 ± 1.0	5.00 ± 0.13	(1)^a^
200 μm 26%	48.8 ± 1.0	4.25 ± 0.10	0.85 ± 0.01
400 μm 26%	51.8 ± 1.0	4.54 ± 0.11	0.91 ± 0.01
200 μm 43%	49.7 ± 0.9	5.57 ± 0.14	1.11 ± 0.01
200 μm 9%	56.1 ± 1.4	5.83 ± 0.21	1.17 ± 0.01
Ti-felt	50.2 ± 1.2	2.76 ± 0.05	0.55 ± 0.01

a Per def.

Furthermore, the CL activity was characterized at a given *iR*-free cell voltage of 1.42 V resulting in very similar values for all the 2D-PTLs in the range of 5.1 ± 0.6 mA/cm^2^. From the activity, it is possible to estimate the (kinetic) relative CL utilization factor *X*_kin_, as done by Schuler et al.:^8^
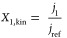
3

The activity value for the 100 μm 26% sample was taken as a reference (*j*_ref_), and the CL utilization *X*_kin_ was estimated for the 2D-PTLs and the Ti-felt from this value. The CL utilization *X*_kin_ of all PTLs is summarized in the form of a bar plot in Figure 2d. For all the 2D-PTLs, we observe close values for *X*_kin_ despite the considerable differences in porosity, pore size, and SPEL.

For instance, assuming that only the regions at the SPEL are active, we would expect that the PTL 100 μm 26% would lead to a 4–5 times increase in *X*_kin_ compared to the 200 μm 9%. Similarly, if we assume that the regions under the PTL land are not accessible and not utilized, we would expect a severe decrease in *X*_kin_ when comparing the 2D PTL with 43 and 9% porosity (or 57 and 91% PTL land). However, since the experimentally obtained utilization values are nearly identical, we can assume that the CLs in each case are equally and almost fully utilized at low current densities. At higher current densities, mass transport and ohmic losses start to dominate the performance and so it is difficult to make a similar statement regarding CL utilization. In the high current density regime, we can expect major fractions of the CL to become inactive, either by limitations in electronic or mass transport.

Furthermore, it is also evident that for the 2D-PTLs, the CL utilization is two times higher in comparison to the reference Ti-felt, which we attribute to its perfectly smooth surface which maximizes electrical contact with the CL and minimizes possible damage. In Figure S1b, a post-test CCM of the 2D PTLs is shown, which shows homogeneous contact and low damages of the CL, especially in comparison to typical deformations and cracks observed for Ti-felt shown by Schuler et al.^8^

#### 2.2.2 HFR and Overpotential Breakdown

Figure S3 shows the *iR*-free cell voltage, as well as the kinetic, ohmic, and mass transport overpotentials, as a function of current density. As expected from the Tafel slopes, only negligible differences (<20 mV) are observed from kinetic losses. The mass transport losses and the ohmic losses exhibit major differences among the different 2D-PTLs with the same trends with porosity, pore size, and SPEL as observed in the overall performance in the previous section.

The high-frequency resistance (HFR) dependence with current density is plotted in Figure S4, where we can also observe similar trends as for the cell voltage. The Ti-felt features a slight decrease in HFR which can be well explained by the through-plane heat resistance of the PTL. Increasing power density causes a local increase in temperature, which can lead to a reduction in the measured HFR.^8,17^ However, for all the 2D PTLs, we observe a strong increase in HFR with increasing current density. This increase is not equal for all materials but follows again the same trend (porosity, pore size, and SPEL). The reason for the increase in the HFR is most probably related to a local dry-out of the membrane below the PTL land (contact areas), where water transport is occurring solely via the open porosity of the CL. Although we do not have ultimate proof that this effect is related to membrane dry-out, we can exclude other possible reasons like degradation or thermal effects. Furthermore, this hypothesis is supported by continuum modeling results, presented in Section 2.4. The differences in HFR at low current density are caused by differences in contact resistance between the used 2D PTLs, despite the equal acid treatment for all PTLs. In our particular case, we did not use PGM coating at the PTL interface since commercial CCMs with high Ir-loadings were used. Although the interfacial resistance is less pronounced at these loadings, there is still an impact when the PTL is not coated with PGM, as observed previously by Kang et al.^49^ which is something that need to be considered. Nevertheless, the interesting trend in HFR at increasing current densities that we observed with the different 2D-PTL structures represents a piece of useful information for further understanding of how the interface affects CL utilization.

### 2.3 Determining the Active Region

This section aims to quantify how well the CL is utilized as a function of current density and interface properties. Using a combination of analytical modeling and the information obtained from the electrochemical analysis, we predict the utilization of the CL in different regions.

#### 2.3.1 Relative CL Utilization Using HFR

**Figure 3:**
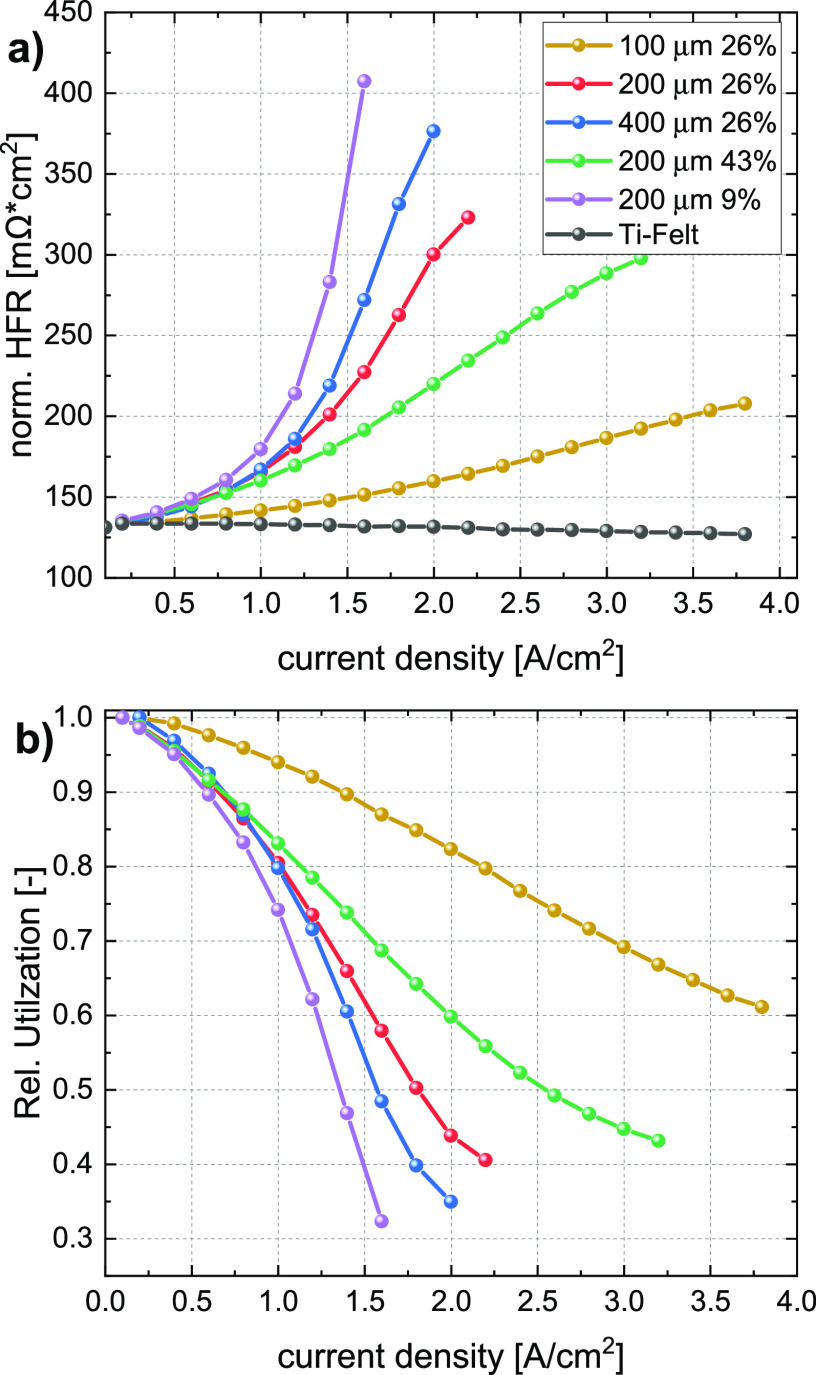
(a) Normalized high-frequency resistance is taken as a base to calculate and (b) the relative CL utilization *X_j_* in dependence of current density (eq. 4).

If we discard the differences in contact resistance between the 2D PTLs and normalize the HFR to an equal starting point at low current density (0.1 A/cm^2^), we can think of the HFR increase with current density as a measure of the reduction of utilized area. Assuming that the Ti-felt leads to continuous, constant utilization of the CL (regardless of the current), we can take this as a baseline to quantify the CL utilization as a function of the current density. The reasoning behind this lies in the fact that the HFR depends on the normalized surface area. If the HFR [mOhm × cm^2^] is increasing due to partial membrane dry-out, the usable surface area will consequently decrease. With these assumptions, we can then calculate the relative utilization of the CL at each current density step assuming that this is reflected in the proportional increase in the HFR. In Figure 3a, the HFR normalized to the same starting point of the Ti-felt’s HFR is shown, discarding the differences in contact resistances and only considering the impact of current density. We can observe that depending on the structure, the HFR increases up to a factor of 4 from the base HFR (Ti-felt), with the same structural trend as observed for the performance in Section 2.2.1. We can translate the relative increase in HFR to a relative utilization as a function of current density (*X*_j_) using the following relationship:
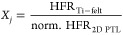
4

From this, we can obtain the respective relative CL utilization as a function of current density *X*_j_ for each 2D-PTL, as shown in Figure 3b. Taking the example of the 2D-PTL with 200 μm pores and 26% porosity, we can observe that at ∼1.8 A/cm^2^, the HFR is roughly two times higher, resulting in a relative utilization of only 50%. This calculation shows that the choice of interface structure can have a crucial impact on the efficient utilization of the catalyst and hence on the overall performance.

#### 2.3.2 Estimating the Penetration Depth under the PTL Land

The information on CL utilization provided in the previous section can be used to better understand the transport mechanism at the PTL/CL interface and in the CL. To validate the assumption of HFR increase as information on the lost area, we corrected the polarization curve by adapting the current density to a normalized area based on the respective CL that is utilized:
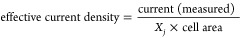
5

**Figure 4:**
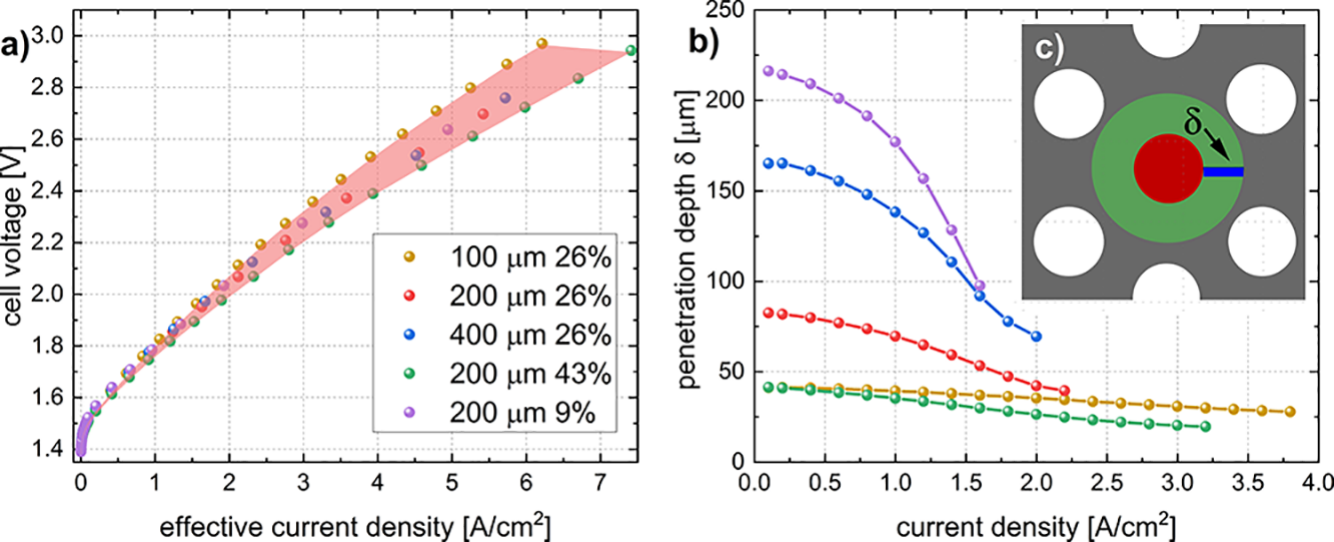
(a) Corrected polarization curves based on the relative CL utilization according to formula 4, (b) estimated IP penetration depth of water under the PTL land based on eq. 5, and (c) sketch of the pore arrangement with the reachable penetration depth δ_*j*_ as a blue line.

Using this correction, we obtain the “effective” polarization curve for the remaining active area, as shown in Figure 4a, where we can observe that the performance for all 2D-PTLs are now nearly identical (specifically when comparing them to the polarization curve in Figure 2a), showing no trend in dependence of the interface properties. This seems to confirm that the difference in performance observed in Figure 2 is mainly caused by the loss in active area which is reflected by the HFR increase. We attribute this loss of accessible surface area under the lands mainly to membrane dry-out due to the poor accessibility of water via the open porosity of the CL.

Having this information available, we can go a step further and calculate the reachability of water in the CL under the PTL land. This reachability or penetration depth δ_j_ is the effective maximum distance of water transport in the IP direction from the pore edge to the area of the CL under the PTL land, at a given current density. Figure 4c shows a sketch of pore arrangement with the penetration depth sketched as a blue line. This value can be estimated by simplifying the problem into two different limiting cases: (i) full utilization of the open pore spaces, meaning that IP electronic conductivity is not limiting CL utilization, or (ii) open pore space is not utilized, meaning that the high electronic resistance of the CL hinders the electrochemical reaction with the consequence of not utilizing the pores. We calculated the penetration depth δ_j_ for both cases and found that for scenario (i) the penetration depth for some PTL/current combinations reaches negative values, indicating that this assumption is not correct. The calculation and more information on the used formula can be found in the Supporting Information in Figure S5. For assumption (ii), meaning poor open pore space utilization, we can calculate the penetration depth δ_*j*_ as follows:
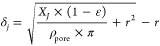
6

where *X*_j_ represents the CL utilization as a function of current density, ε denotes the porosity of the 2D-PTL, ρ_pore_ represents the pore density per area, and *r* is the pore radius of the respective PTL. The calculated penetration depth δ_j_ as a function of current density is shown in Figure 4b. At low current densities, we can observe different penetration depths for each PTL structure, which depend on the radius, the porosity, and the respective pore density. For instance, for the sample with 200 μm pores and 9% porosity, the initial penetration depth is relatively large since CL utilization at low current density is high. However, with increasing current density, i.e., increasing oxygen production rate, the penetration depth decreases strongly for samples with low SPEL/pore density. For the 2D-PTLs with high SPEL (e.g., 200 μm 43% and 100 μm 26%), the initial values for δ_j_ are lower, due to the high pore density. The same applies to the behavior with increasing current density, where we observe a smaller drop in penetration depth by only ∼50%. In fact, we can observe that all materials have a different rate of penetration depth loss, but judging from the extrapolation of the curves, we can see that they all seem to converge toward the same limiting value for δ_*j*_ of roughly ∼20–25 μm at high current densities, regardless of the pore size and porosity. This indicates that the mass transport in the CL under the PTL land, for the present CL structure with a thickness of about 10 μm, is limited to a reachability of ∼20–25 μm from the pore edge, regardless of the interfacial structure. We assume that this value will vary with CL thickness (less at lower thickness) and porosity (less at lower porosity). However, we can generally conclude that when translating these findings from model structures to real PTLs (e.g., fiber or sintered particles) “inactive area limitations” due to poor transport in the CL should not occur if the particle or fiber radius is below ∼20–25 μm. Since most commercially available materials (e.g., Bekaert, Mott) have smaller particle/fiber radius, these limitations are usually not observed for standard catalyst loadings but might become relevant when lower Ir-loadings (i.e., thinner CLs) are used. In general, this finding suggests that smaller particle sizes, i.e., lower needed IP penetration depth, are favored. However, this analysis is based on the assumption of poor open pore space utilization, i.e., case (ii) of the above assumption. The validity of this assumption is analyzed and discussed in the following section.

#### 2.3.3 Calculating the Activity in the Open Pore Spaces

For the calculation of the penetration depth in the previous section, we assumed that the CL at the open poor spaces is not utilized. To address this assumption, we apply the principle of electronic charge conservation to analytically investigate the CL utilization at the open pore spaces of the interface.

The regions of the CL beneath the open pore spaces have good access to water supply and oxygen removal due to the unimpeded pore volume. Hence, the limiting factor in this region is the electronic resistance of the CL, which can be impaired due to the tortuosity of the percolated network. In particular, at uncompressed regions of the CL (i.e., under the open pore spaces), the effect can be aggravated by ionomer swelling under wet conditions, as shown by Schuler et al.^30^ The poor electronic conductivity of the CL affects the electrochemical reaction by a gradual potential drop across the open pore. This potential drop is effectively a nonlinear reduction of the overpotential along the cross-section of the pore. Assuming the OER overpotential follows the Tafel behavior, the potential inside of the pore ϕ(*r*) can be calculated by solving the following ordinary differential equation:
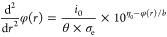
7

where ϕ(*r*) is the radially varying electronic potential, *i*_0_ the exchange current density (0.6 × 10^–9^ A/cm_Ir_^2^),^50^ θ is the CL thickness (∼10 μm), σ_e_ is the CL electrical IP conductivity (∼0.1 S/cm),^30^ η_0_ is the nominal OER overpotential (i.e., given by the current density at the pore edge, before voltage drop), and *b* is the Tafel slope. A sketch, as well as more details, can be found in the Supporting Information (Figure S6).

**Figure 5:**
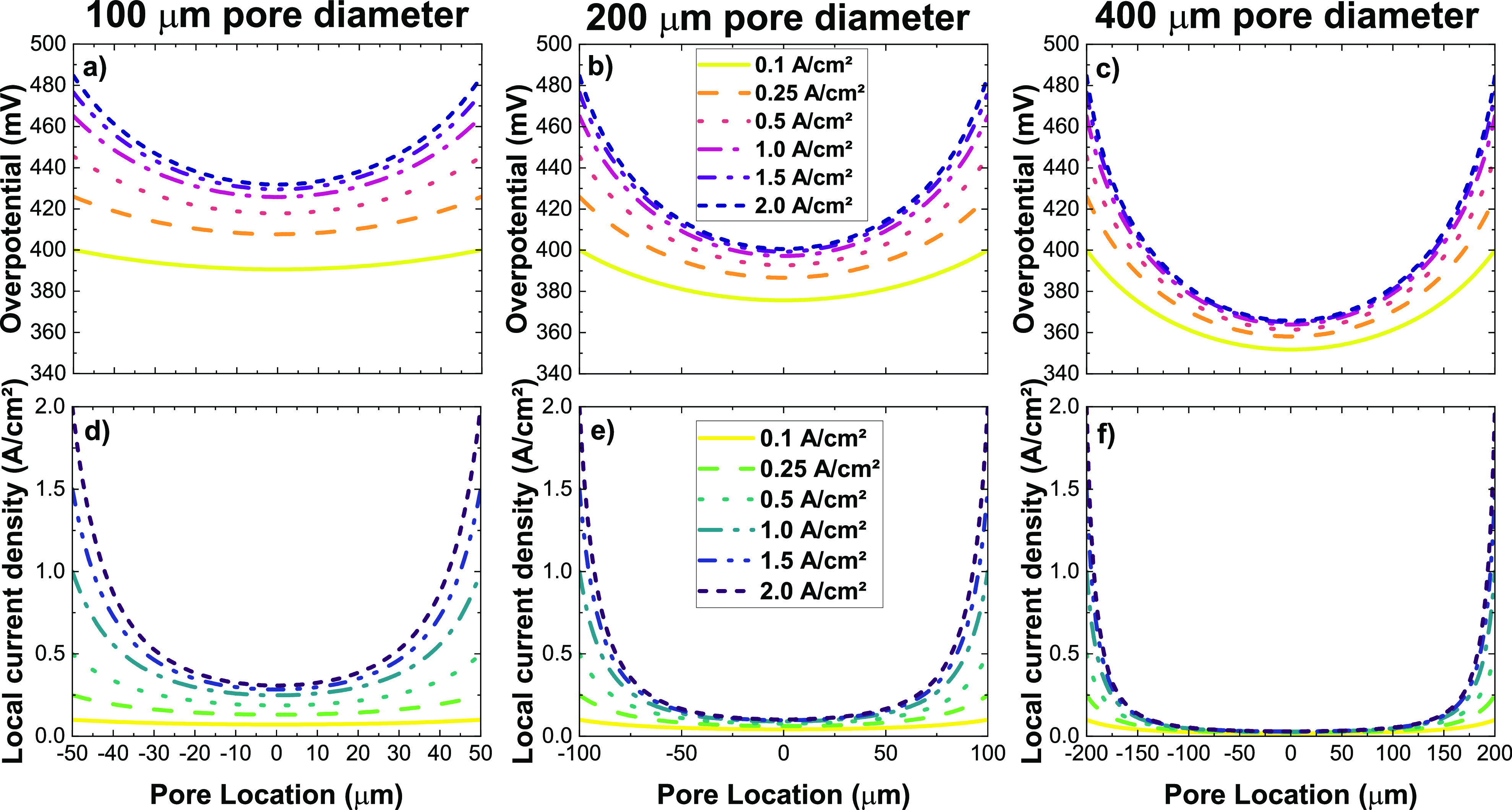
Calculated overpotential drop (a–c) and respective local current density (d–f) according to eq 7 for the three different pore sizes (a, d) 100 μm, (b, e) 200 μm, and (e, f) 400 μm used in this study and a selection of nominal current densities.

In Figure 5a–c, the calculated overpotential drop for different nominal current densities (at the pore edges) from 0.1 to 2 A/cm^2^ is shown for the three different pore sizes in our PTL matrix. There is a fast drop of the overpotential from the pore edge with a minimum in the middle of the pore (pore location = 0). The shape of the curves is similar for all pore sizes and current densities, but with an increasing potential drop as the current density and the pore size increase. For instance, at a current density of 1 A/cm^2^, the potential drop from edge to center is in the range of 20, 50, and 80 mV for 100, 200, and 400 μm pore diameters, respectively. To put this potential drop into perspective, we can calculate the local current density at the position *r* from the pore edge according to the Tafel model:
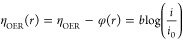
8

The results are plotted for the three pore sizes in Figure 5d–f, where we can observe high losses in local current density. Already for the smaller pore sizes, the drop in current density is very high, meaning that most of the area at the open pore space is not utilized due to the high IP resistance of the CL. Furthermore, by integrating the current densities across the pores, we have estimated an average current density in the open pore of 0.41, 0.23, and 0.12 A/cm^2^ for 100, 200, and 400 μm, respectively, at a nominal current density of 1 A/cm^2^. Overall, we can assume that the open pore spaces are only poorly utilized and the previous assumption of inactivity of the pore, for the calculation of the penetration depth δ_j_ under the PTL, is valid. Not only does this have a negative impact on cell performance, but large changes in the local electrochemical conditions may also lead to an increase in electrochemical degradation of the CL.

### 2.4 3D Multiphysics Modeling: Current Distribution, Membrane Hydration and Gas Phase in the CL

To investigate the hypotheses of Sections 2.2 and 2.3, a 3D multiphysics model was developed based on continuum transport phenomena. This model is an extension of the 2D model developed previously.^48^ A full description of the model is given in the reference, and a brief description is given in Table 3. These equations allow us to visualize the distribution of water in both the CL and the membrane under the lands and open pores of the 2D-PTL.

**Table 3:** Description of Dependent Variables and Governing Equations Used in the 3D Multiphysics Model

dependent variable	description	domains present	governing equation(s)
ϕ_A_	anode electronic potential	anode flow plate, PTL, anode catalyst layer (ACL)	charge conservation + Ohm’s law
ϕ_C_	cathode electronic potential	anode flow plate, GDL, cathode catalyst layer (CCL)	charge conservation + Ohm’s law
ϕ_I_	ionomer potential	ACL, membrane, CCL	charge conservation + Ohm’s law
*f*_w_	water mass fraction in ionomer	ACL, membrane, CCL	mass balance including diffusion + electro-osmotic drag
*p*_G_	gas phase pressure	ACL, CCL	Darcy’s law
*p*_L_	liquid phase pressure	ACL, CCL	Darcy’s law

**Figure 6:**
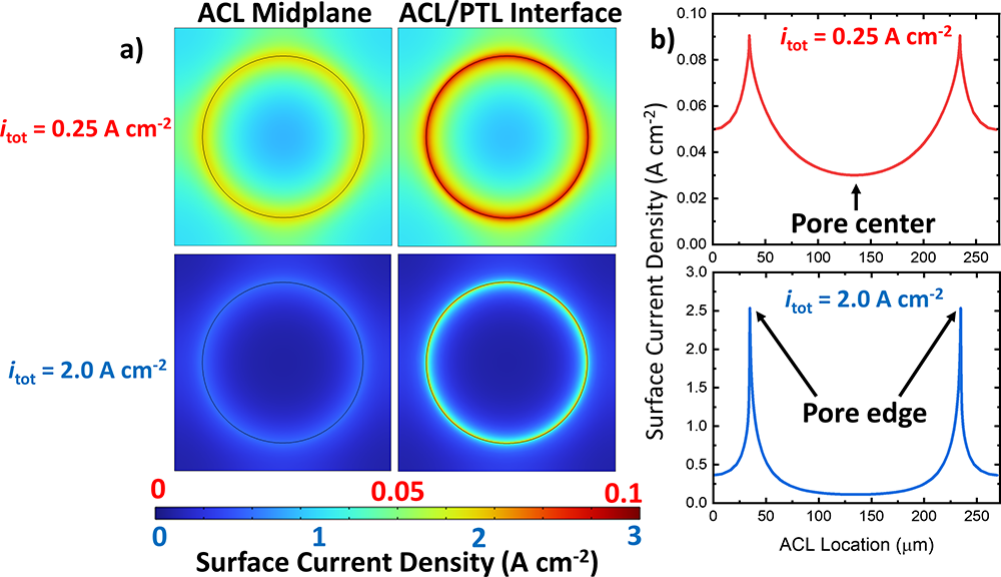
(a) 2D and (b) 1D maps of the surface current density at total cell currents of 0.25 and 2.0 A cm^–2^ for a 200 μm pore.

Figure 6a shows the distribution of surface current density in the anodic CL beneath the 2D-PTL. 2D plots are shown at the CL/PTL interface and at the midplane of the ACL (half of the CL thickness in the through-plane direction). For *i*_tot_ = 0.25 A/cm^2^, we can see that there is still some utilization under the PTL lands, as indicated by the lighter blue and green colors. However, at 2.0 A/cm^2^, we can observe that there is essentially no utilization under the lands, indicated by the uniform dark blue color. Figure 6b shows 1D cross sections of the surface current density at the CL/PTL interface, in which the visible spikes correspond to the observed maximum at the pore edges that was predicted in Figure 5. These results corroborate the reasoning in Sections 2.2 and 2.3. In Figure S7 of the Supplementary Information, the 2D maps and the respective 1D cross sections for the normalized OER surface current are shown. Here, the surface current density is normalized to the maximum surface current density to show the relative spatial variation of the OER.

**Figure 7:**
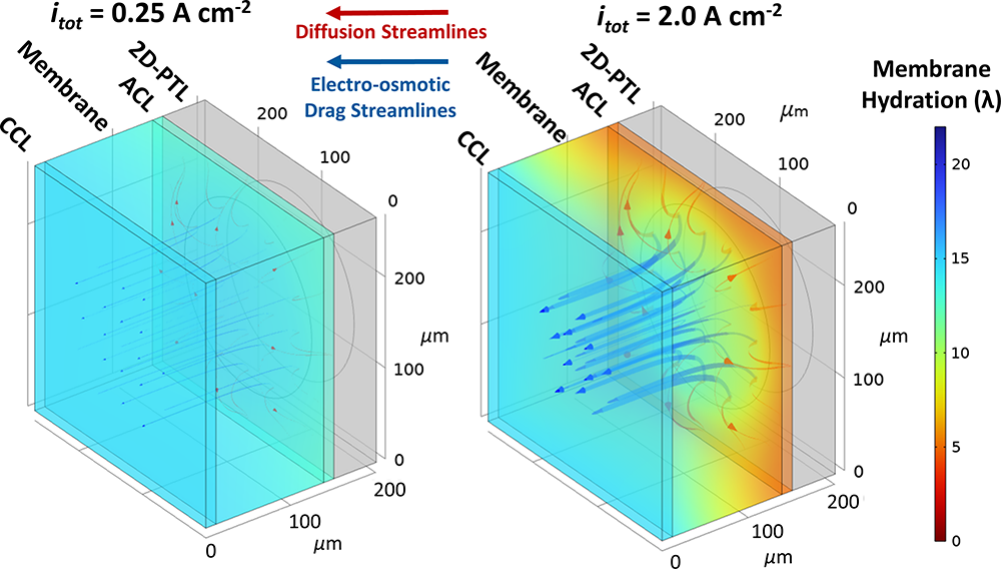
3D visualization of membrane hydration in low and high current density regimes for a 200 μm pore.

Figure 7 shows a 3D visualization of the membrane and CLs hydration underneath the pore of the 2D-PTL in the low and high current density regimes. Flux streamlines are also shown that isolate the contributions from diffusion and electro-osmotic drag. The widths of the streamlines are proportional to the local magnitude of the flux term. Diffusion streamlines follow the gradients in local water content, while electro-osmotic drag streamlines essentially point linearly from anode to cathode, following the ionic current. At 0.25 A/cm^–2^, the membrane is uniformly hydrated, and the contributions from diffusion and electro-osmotic drag are roughly the same magnitude. However, at 2.0 A/cm^2^, it can be seen that the membrane dehydrates under the PTL lands. This can be explained by the difference in magnitude between the diffusion and electro-osmotic drag contributions to the net water flux. At high currents, electro-osmotic drag rapidly pulls water away from the 2D-PTL pore. Diffusion is the only mechanism by which water can reach under the lands. At high currents, the electro-osmotic drag flux becomes orders of magnitude greater than the diffusive flux, and the regions under the lands dry out. In other words, Figure 7 represents a quantitative visualization of the water penetration depth, as depicted in Figure 4b. Here, we also see that the water reachability phenomenon also extends into the bulk membrane.

**Figure 8:**
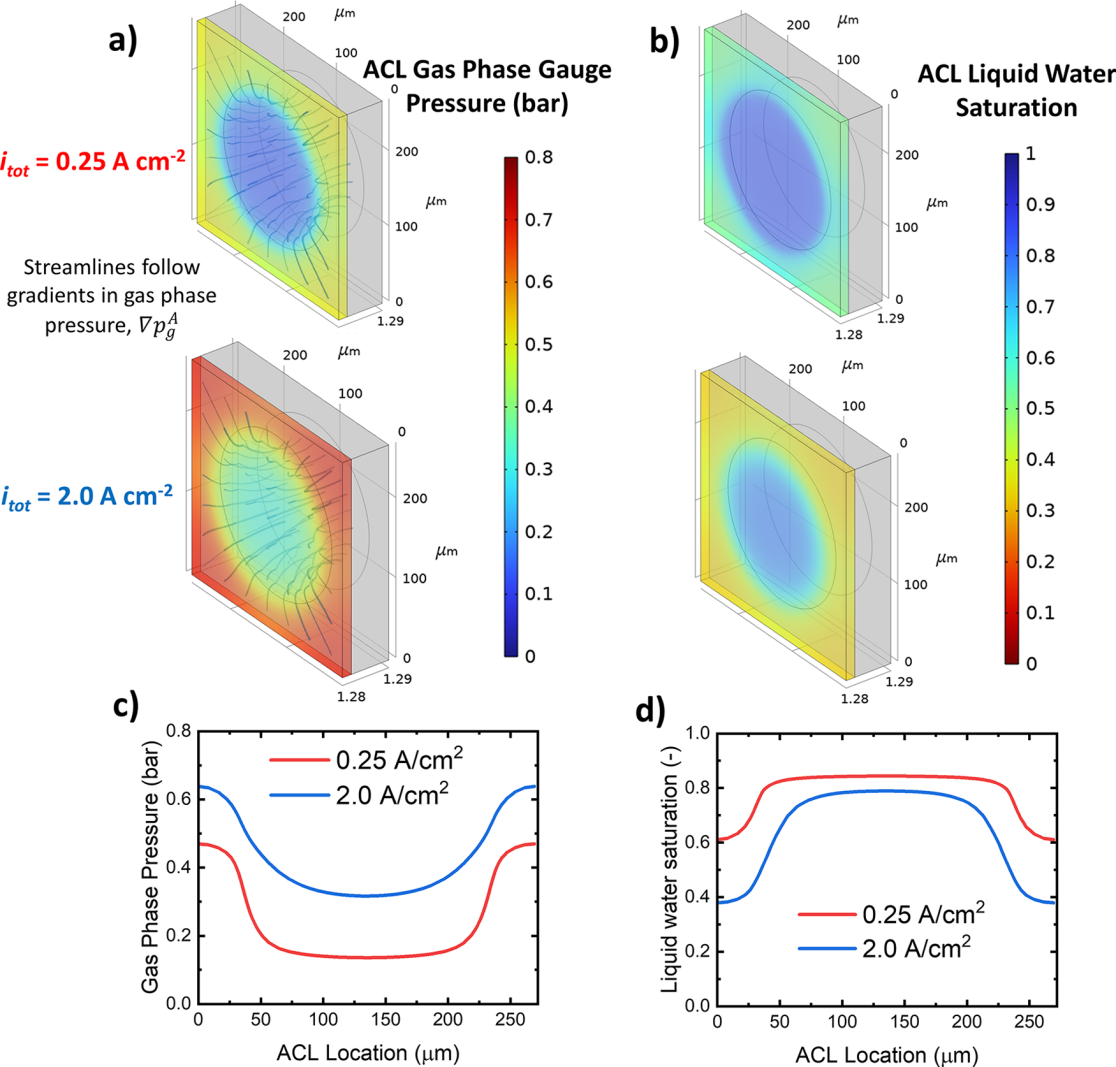
3D visualization of the (a) anodic CL gas phase gauge pressure and (b) ACL liquid water saturation for low and high current densities. 1D cross sections of the (c) gas phase gauge pressure and (d) the liquid water saturation.

The water reachability can be explained well by Figure 8a, which shows a 3D visualization of the gas phase gauge pressure distribution in the ACL for low and high current densities. The displayed streamlines follow the gradients in gas phase pressure. We observe how the gas phase pressure builds up below the PTL land while at the pore space location, the gauge pressure in the ACL stays relatively low. This effect is obviously more pronounced at high current densities due to the higher oxygen evolution rate. The consequence of the gas buildup can be observed in the ACL distribution of the liquid water saturation, as shown in Figure 8b. The pore regions are highly saturated with water while under the PTL land, we observe a strong decrease in liquid saturation. For a better understanding of the location dependence, a 1D cross-section of the gas phase gauge pressure and liquid water saturation is shown in Figure 8c,d, respectively. At the PTL land region, we need to consider that vapor phase transport is of increasing relevance, as we will discuss in the next section.

### 2.5 Pressure Dependence and Discussion on the Transport Mechanism

**Figure 9:**
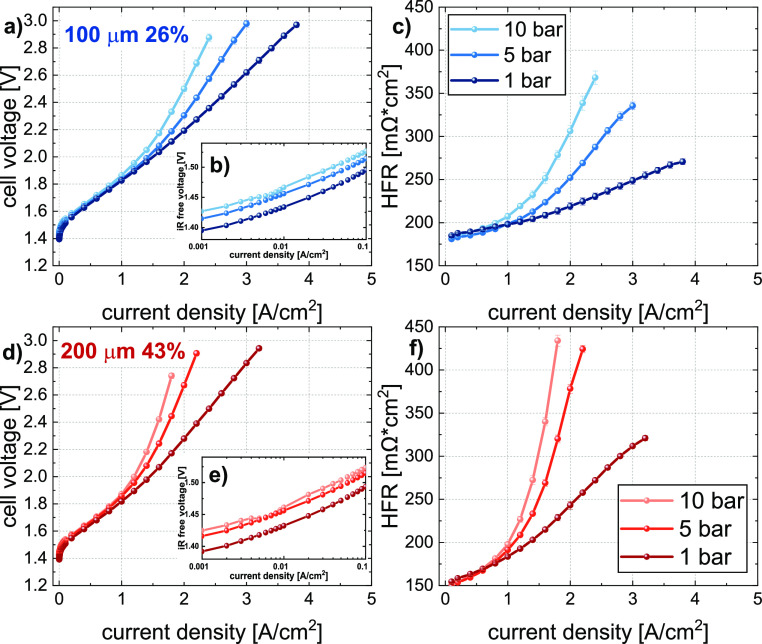
Pressure dependency of the (a, d) polarization curve, (b, e) the kinetic region, and (c, f) the measured HFR for two exemplary samples of 100 μm 26% (a–c) and 200 μm 43% (d–f) measured at 80 ° C.

In Figure 9, the dependence of the polarization curve on gas pressure and the respective HFR trend of two distinctive 2D-PTL samples (100 μm 26% and 200 μm 43%), are shown. A strong trend is observed with increasing current density, showing that increasing gas pressures lead to severe losses in performance, as well as to a significant increase in the HFR. The magnitude of this effect is similar for all the 2D-PTL samples, showing that there is a clear dependency of the ohmic and mass transport losses on gas pressure. According to the Nernst equation, with increasing gas pressure, a slight increase in the thermoneutral cell voltage is expected, which can also clearly be seen in the low current density range, as shown in Figure 9b, e. However, the strong deviations that start to arise at current densities >1 A/cm^2^, cannot be explained by the difference in thermoneutral cell voltage. Following the reasoning of the last subchapters and together with the strong HFR increase, we can confidently say that the mass transport in the CL under the PTL becomes more limited with increasing gas pressure. Because liquid water has two roles in this system (both hydrating the membrane and acting as the reactant in the OER), we see the impact of this effect in both the HFR and the *iR*-free voltage.

This effect can be explained if we consider that the transport in the CL (at least under the PTL land) is at least partly occurring in the vapor phase. The diffusion coefficient of water vapor (D_H2O, (v)_) is inversely proportional to the pressure:
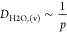
9

This means that with increasing pressure, diffusional transport of water to the reactant site is slowed down, which leads to an earlier dry out of the membrane at that respective area. Therefore, we hypothesize that water is transported in the liquid phase in the PTL. At the interface between the PTL and CL at least part of the pores are gas-filled and (part) of the water changes from liquid to the vapor phase. At high current densities, oxygen gas is evolved at a large volumetric rate. As the gas pressure increases, so does the local capillary pressure (*p*_C_ = *p*_gas_ – *p*_liq_), which leads to lower liquid water saturation in the CL, as we could observe in Figure 8. In previous work on 2D-PTLs, Wrubel et al.^48^ referred to this effect as “gas blinding” and noticed a significant decrease in liquid water saturation underneath the lands of the 2D-PTL. This not only leads to an increase in HFR (due to lower water uptake from the vapor phase compared to the liquid phase) but in severe cases can also lead to mass transport limitations in the OER. At an operating temperature of 80 °C, water will have a saturation pressure of 47 kPa. For the short distances of the through-plane direction of the CL (1–10 μm), such a local vapor feed can sustain significant current densities. However, for the longer IP distances (10 to 100 s micrometers) vapor transport becomes insufficient and limits current density, leading to the above-described phenomenon of the reduced active area. The observed pattern of HFR increase with current density is similar to the one that has been observed in vapor-fed electrolysis^50^ (although at different magnitudes). On the other hand, other studies have suggested that the generated oxygen could also be transported as dissolved oxygen in water by diffusion or convection.^51^ Therefore, further studies would be needed to strengthen this hypothesis of vapor phase transport.

## 3 Conclusions

In this study, we have designed and manufactured two-dimensional PTLs by precise laser drilling of thin Ti-sheets. A matrix of five PTLs with different pore sizes, porosities, and inter-pore distances was tested in a PEWE cell for in-depth electrochemical analysis, with the aim of better understanding the role of the PTL/CL interface geometry on PEWE performance and CL utilization.

The kinetic analysis (low current densities) showed improved CL utilization with 2D-PTLs, as compared to standard Ti-fiber materials, probably related to the smoother surface. Between the different 2D-PTLs, no significant difference was observed, showing that at low current densities (before ohmic and mass transport losses become relevant), CL utilization is not affected by the properties of the 2D-PTL/CL interface. At high current densities, however, a significant decrease in performance and a severe increase in the HFR were observed. Smaller pore sizes and higher porosity showed better performance and a general correlation of performance with specific pore edge length was observed.

The increase in HFR, attributed to membrane dry-out, was used as an indicator for reduced CL utilization and thus reduced active area. The relation between the HFR increase and the loss of the utilized area was validated by polarization curves normalized by the utilized area. Based on a simple geometric model, we found that at high current densities (i.e. >2 A/cm^2^), for the employed (commercial) anode catalyst layer, current is limited to a maximum of ∼20–25 μm from the pore edge under the PTL land, regardless of the interfacial structure. For this, it was assumed that the pore space is poorly utilized, which we supported by modeling the open pore space utilization based on the voltage drop due to poor IP electronic conductivity.

To better understand the water distribution under the PTL lands, a 3D multiphysics model was used to calculate the current distribution and predict the membrane dry-out, which is a major cause of performance losses with these structures. Finally, a strong pressure dependence of the CL utilization and mass transport losses was observed. Our findings indicate that the transport of water in the CL under the PTL land is at least partly occurring in vapor form, due to gas blinding from oxygen generation.

Our work with these model interfacial systems sheds light on the transport phenomena occurring at the interface between the PTL and CL and into the CL itself. The findings obtained can be applied to improve the interface properties of available PTLs, where we propose small particle size (for a high-loading CL lower than 20 μm radius) for reducing transport losses and dry-out of the membrane. We also suggest the use of PTLs with small pore sizes to improve CL utilization by minimizing the IP electronic path in the CL. Therefore, the introduction of fine microporous layers with a small particle size of <15 μm and pore size in the range of 5–10 μm is recommended and will be investigated in future studies.

## 4 Experimental Section

### 4.1 Design of Porous Transport Layers with Well-Defined Features

In this work, precise and controllable two-dimensional porous transport layers were used as an additional layer placed at the interface between a standard Ti-fiber PTL and the CL. These model PTLs referred to as 2D-PTLs were fabricated by precise laser drilling of thin Ti-sheets (140 μm nominal thickness, 99.6% + titanium), which was commissioned to Felastec AG (Switzerland). The practical limitation for laser drilling sizes on titanium of this thickness and necessary pore–pore distance was found to be in the range of 100 μm, to achieve a suitable porosity. While smaller drillings are in principle possible, the removal and cleaning of the material in the holes after laser drilling are currently not possible for the required hole/pore density necessary to achieve sufficient porosity. The porosity is given by the surface pore density, which is controlled by the distances between the pores. With this specification, a matrix of five 2D-PTLs with three different pore sizes (at equal porosity of 26% and 100, 200, and 400 μm pore diameters) and three different porosities (at constant pore size of 200 μm and 9, 26, and 43%) were designed and are summarized in Table 1.

### 4.2 X-ray Tomographic Microscopy (XTM): Data Acquisition and Image Processing

The X-ray tomographic images were acquired using a lab-CT scanner phoenix nanotom m (General Electric, Germany). 2 × 4 mm^2^ samples were mounted perpendicular to the beam. The acquisition parameters were set to 90 kV and 280 μA and a voxel cube edge length of 2 μm was chosen. We acquired 1500 projections over 360° and 1500 ms exposure time (per projection), resulting in a scan time of approximately 40 min. A region of interest of 1800 μm × 1800 μm × 140 μm was selected from the middle of the sample to avoid edge effects. From the tomographic data, gray-scale images were segmented manually selecting an appropriate thresholding value. The images were processed in the open software ImageJ, subsequently, the isosurface was calculated using in-house MATLAB scripts, and the surface renderings were prepared with Paraview software.

### 4.3 Electrochemical Performance

#### 4.3.1 PEWE Cell and Test Bench

The electrochemical measurements of the cells with 2D-PTLs were carried out using in-house developed cells with an active area of 20 × 20 mm^2^. Commercial catalyst-coated membranes (CCMs) from Greenerity GmbH (Germany, E400 Gen. 3, Batch: # 0878-20) with Ir-based catalyst on the anode and Pt/C catalyst on the cathode coated on a Nafion 115 membrane (127 μm nominal dry thickness) were used for all measurements. On the cathode side, a 1 mm Ti-fiber PTL (Bekaert, 56% porosity) was used in all measurements, while the interface anodic 2D-PTL was varied. Due to the two-dimensional nature of the interface PTLs, a Ti-Fiber PTL (Bekaert) with high porosity (76%) was used as a distribution layer on the flow field side at the anode. The oxide passivation layer on the Ti-PTLs was removed/minimized by an acid etching^52^ step in 2 mol/L aqueous HCl for 20 min at room temperature. This was followed by rigorous rinsing with de-ionized water for removing residual acid and four 15 min ultrasonication bath steps in deionized water, 50 vol % acetone/water, 50 vol % ethanol/water, and deionized water again. Two PTFE-coated fiberglass gaskets (FIBERFLON, 2 × 130 μm on the cathode and 2 × 60 μm on the anode) were used on each side for gas tightness and electric insulation. The cell includes a spring mechanism that keeps the CCM compression constant (2.5 MPa) and independent of clamping pressures (higher for sealing reasons). The cell has gold-coated flow fields with five parallel channels (1 mm depth, 20 mm length, and 2 mm width) separated by 2 mm ribs. A thermostat and a thermocouple located close to the active area control and measure the cell temperature during operation. DI water is recirculated at the anode side with a volume flow of 30 mL min^–1^ cm^–2^ and through an integrated ion exchange bed for sustaining water purity during performance tests. The in-house developed test bench, as well as more information related to the cell, is described in the references.^8,17,53^

#### 4.3.2 Electrochemical Measurements

The protocols for electrochemical performance tests were analogous to the references.^8,17,53^ A Biologic VSP**-**300 (Bio-Logic SAS, France) potentiostat is used for electrochemical measurements. The potentiostat allows for simultaneous HFR measurements while recording the polarization curves. We conditioned the cells at 5 bar in N_2_(g)/H_2_O(l) for at least 12 h before the potentiostatic break-in cycle protocol (2.0–2.6 V, 50 °C, 10 bar). The measurements were started when stable performance and HFR conditions were reached. We recorded the polarization curves at a current density range of 0.001 to 2–4 A/cm^2^ in galvanostatic mode with holding times of 10 s for each current density step. The final current density range was dependent on the performance, and measurements were stopped at an upper safety voltage limit of 3 V to avoid major cell degradation. The HFR was measured at each step at 25 kHz for 1 s. We recorded polarization curves at 10, 5, and 1 bar (ambient) balanced gas pressures and 50 and 80 °C cell temperature, respectively, and repeated each measurement three times for each PTL, temperature, and pressure. The data shown here are the average of the three measurements. For most Figure, at 1 bar (ambient pressure) and 80 °C results are shown in this manuscript, as similar trends are observed for all conditions. However, the impact of pressure variation (10, 5, and 1 bar) is shown and discussed for two of the samples.

#### 4.3.3 Overpotential Analysis

The total cell voltage can be described as the sum of the reversible cell potential *E*^0^(*p*, *T*) and the three main overpotentials: kinetic (η_kin_), ohmic (η_Ω_), and mass transport overpotential (η_mtx_).
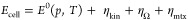
10

The Nernst equation describes the equilibrium cell voltage which depends on the partial pressure of the gases and the operating temperature. The ohmic overpotential can be calculated by measuring the HFR during the recording of the polarization curve
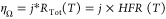
11where *j* is the current density. The kinetic overpotential is then estimated from the Tafel model, assuming a non-polarizable hydrogen evolution reaction, which is governed by the oxygen evolution reaction:
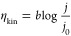
12where the Tafel slope *b* and the apparent exchange current density *j*_0_ are the governing kinetic parameters. The residual overpotential η_mtx_ is typically attributed to mass transport losses (mtx) in the cell, mostly due to the fluidic transport in the PTL and the CL, as well as the ionic transport in the CL.^8,54^ More details on the overpotential analysis are described in reference.^8^

## Supporting Information Available

Scanning electrode microscopy images of 2D-PTLs and post-test CCMs; polarization curves and HFR measurements without the use of support diffusion layer; *iR*-corrected polarization curves and overpotential analysis: kinetic, ohmic, and mass transport losses; measured HFR before normalization; estimated penetration depth and calculation for assumption of full pore space utilization; schematic illustration and detailed explanation on the calculation of the activity in the CL at the open pore spaces; 3D multiphysics model results showing the normalized OER surface current density at low and high total current density (PDF)
