# Supplementary material for: How the Porous Transport Layer Interface Affects Catalyst Utilization and Performance in Polymer Electrolyte Water Electrolysis
Source: ACS Appl Mater Interfaces. 2023 Jul 17;15(29):34750–63. doi: 10.1021/acsami.3c04151 (PMC10375438; doi:10.1021/acsami.3c04151)
Supplement: Supplementary file 1 — am3c04151_si_001.pdf [file am3c04151_si_001.pdf]

Supporting Information:

How the porous transport layer interface affects catalyst utilization and performance in polymer electrolyte water electrolysis

Carl Cesar Weber[1], Jacob A. Wrubel[2], Lorenz Gubler[1], Guido Bender[2], Salvatore De Angelis[1], and Felix N. Büchi[1*]

[1]Electrochemistry Laboratory, Paul Scherrer Institut, 5232 Villigen PSI, Switzerland

[2]National Renewable Energy Laboratory, Golden, Colorado, 80401, USA

*Corresponding author: felix.buechi@psi.ch

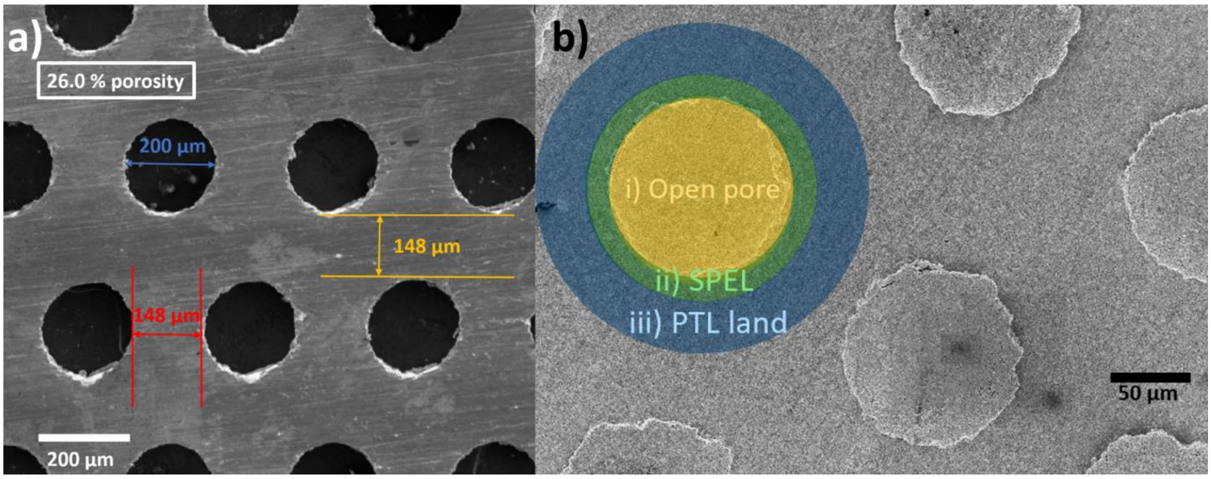

Figure S1. SEM image of a) 2D-PTL with 200 µm pore size and 26 % porosity with the distances sketched and b) post-test CCM tested with a 2D PTL with 100 µm and 26 % porosity with the different interface regions highlighted i) open pore space ii) specific pore edge length (SPEL) or circumference length iii) PTL land (region of the CL under the PTL)

A scanning electrode microscopy (SEM) image of a 2D-PTL with 200 µm pore size and 148 µm x-y spacing (inter pore distance) is shown in Figure S1. Using eq. 1 of the main manuscript this results in a porosity of 26%.

Figure S1b shows a post-test SEM image of the catalyst-coated membrane (CCM) after operation using a 2D-PTL with 100 µm pores and 26% porosity. We can observe different regions in the catalyst layer that are highlighted in colors. The open pore space region, highlighted in yellow, is uncompressed which becomes clear when looking at the edges. In this region, we expect sufficient fluidic transport due to the open porosity but electronic in-plane conductivity might be hindered. The specific pore edge length (SPEL) or circumference of the pore, is highlighted in green (in previous literature this was also referred to as triple phase boundary[1]). These are the regions where open pore space, PTL, and catalyst layer intersect hence where fluidic, electronic, and ionic transport can be ensured respectively[1]. This should not be confused with the triple phase boundary in fuel cell catalyst layer research which is usually the intersection between ionomer, Pt-particles, and pore space. For this reason, we refer to it here as SPEL instead of TPB in order to avoid confusion. Finally, we can observe the smoothly compressed region of the catalyst layer under the PTL land, which is highlighted in blue. In these regions, electronic transport can be ensured through the 2D-PTL but fluidic transport might be less efficient.

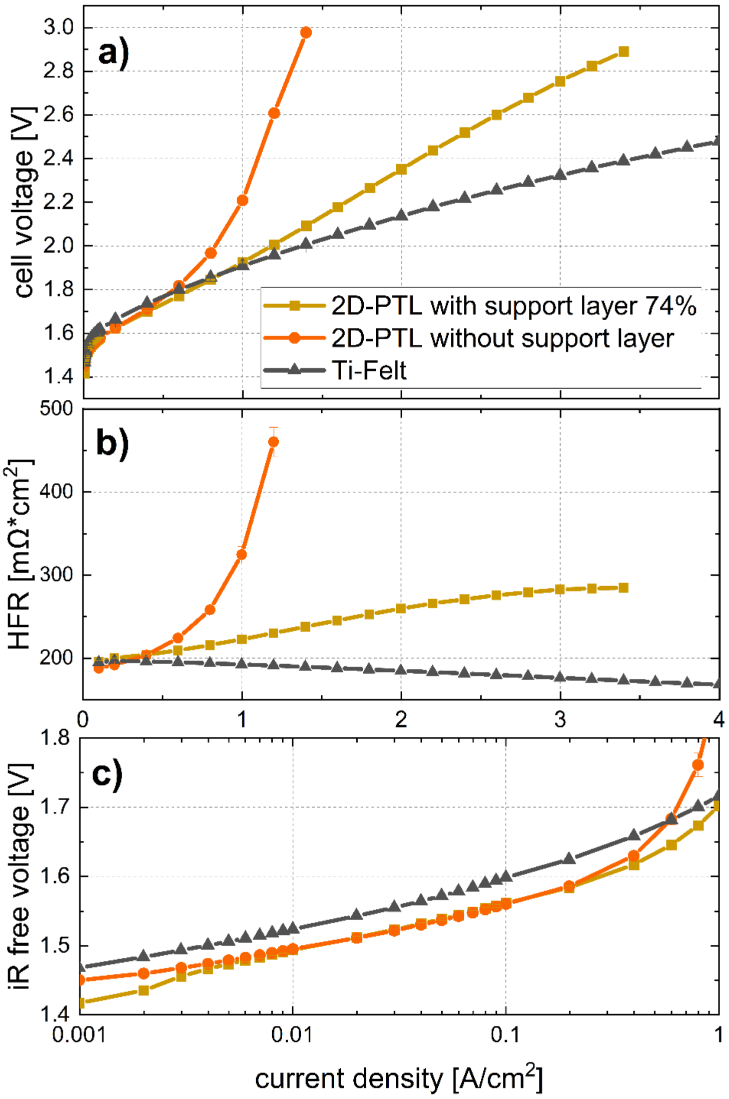

Figure S2. a) Polarization curves and b) HFR measurement comparison using a 2D-PTL (100 µm 26%) with support layer PTL (74% porosity), 2D PTL without SL, and a normal reference Ti-felt PTL c) shows the iR-free cell voltage at the low current density region in a logarithmic scale. Measurements were recorded at 50°C and ambient pressure.

In Figure S2 we can observe the a) polarization curves and the b) the HFR comparison of a 2D-PTL with 100 µm pore size and 26% porosity with a support layer (Ti-Felt 74% porosity), without a support layer and a pure Ti-felt as a reference. The 2D-PTL without the support layer exhibits much higher voltages in comparison to when a support layer is used. Also, the HFR increases at a much higher rate indicating strong membrane dry-out. Figure S2 c) shows the iR-free cell voltages between 0.001 – 1 A/cm^2 in a logarithmic scale from which we can observe a similar (Tafel) slope for all the curves but with a lower intercept for the cells with 2D-PTLs, indicating better kinetic catalyst layer utilization. Furthermore, as mentioned in the main manuscript, we can observe that major losses and differences between all the curves start to arise only at higher current densities between 0.2 – 0.4 A/cm2. Because of the big differences in performance, in the rest of the manuscript a support layer was used when using a 2D-PTL.

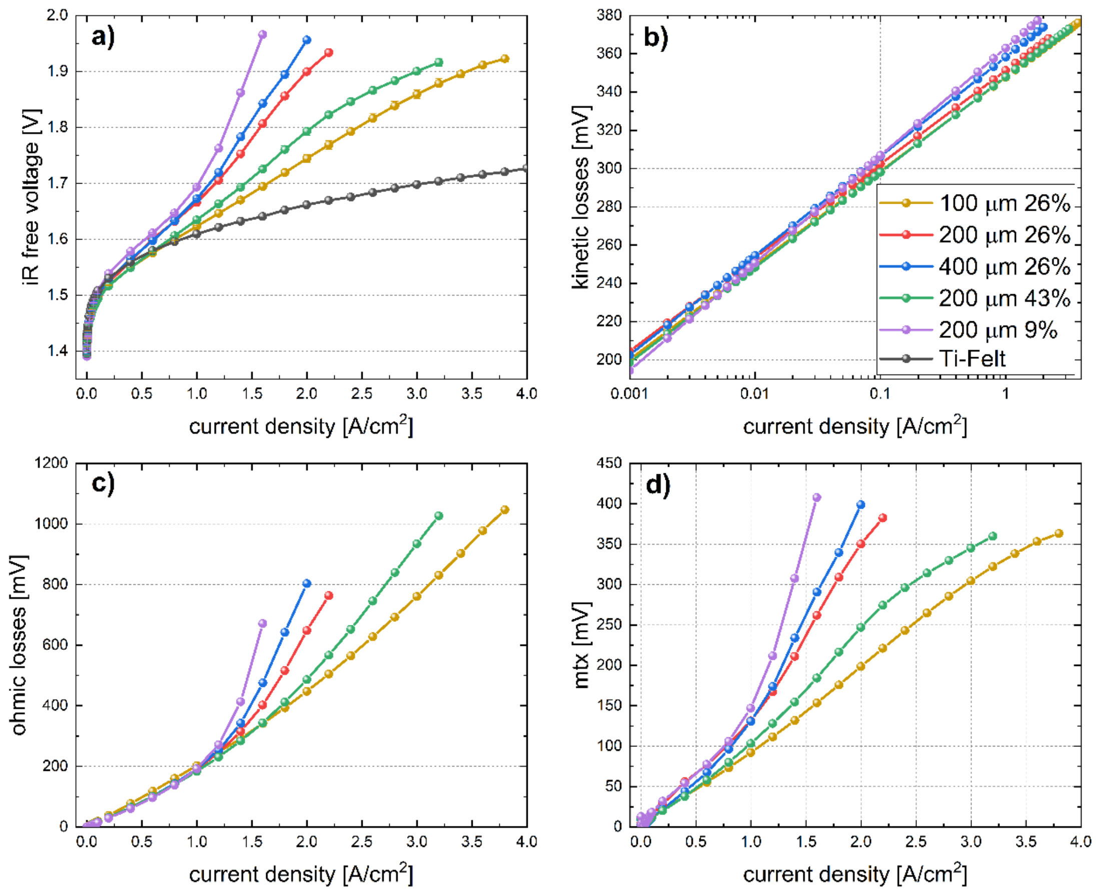

Figure S3. Overpotential analysis of the five 2D-PTLs a) iR-free cell voltage b) kinetic losses by Tafel model c) ohmic losses calculate from HFR measurement and d) mass transport overpotential (mtx) or rest overpotential

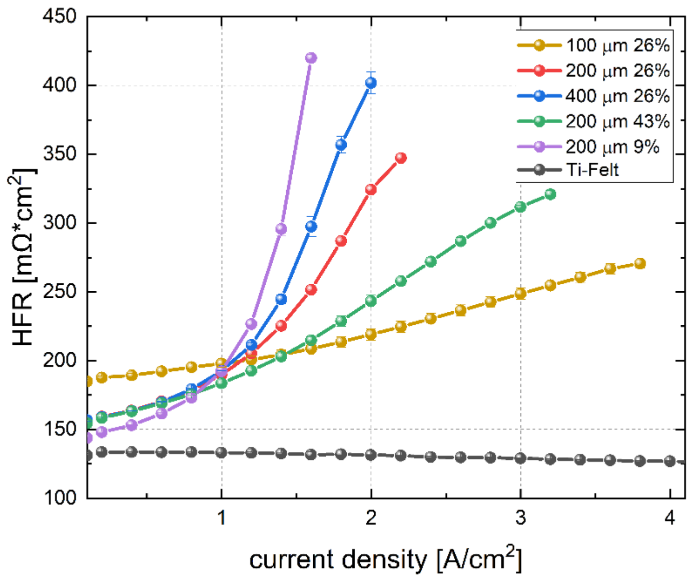

Figure S4. Measured high-frequency resistance for each current density step for the 2D-PTL and the Ti-felt reference before normalization

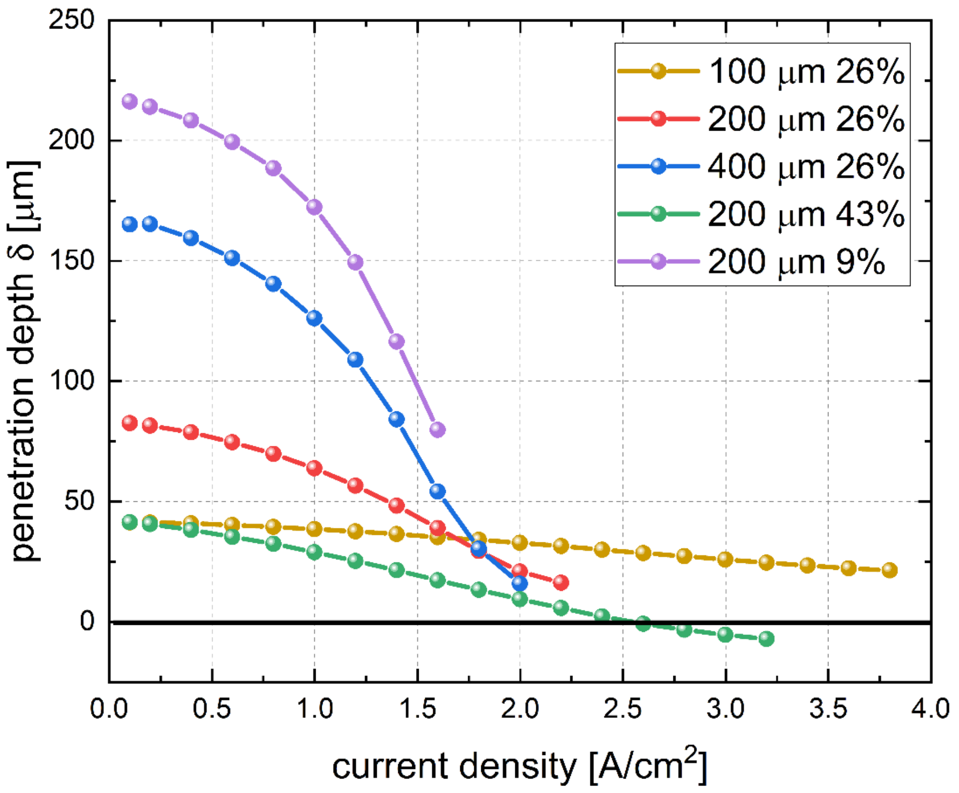

Figure S5. Estimated penetration depth as a function of current density for assumption i) when the open pore spaces are fully utilized

Figure S5 displays the calculated penetration depth as a function of the current density for the assumption i) pore space is fully utilized. The penetration depth for this assumption was calculated using the following formula:

$$\delta_j = \sqrt{\frac{X_J}{\rho_{pore} * \pi}} - r \qquad (1)$$

Where X_j represents the CL utilization in dependence on current density calculated from the HFR using eq. 4 of the main manuscript, ρ_{pore} the pore density (amount of pores per area), and r is the pore radius of the respective PTL. At low current density, the penetration depth is similar to assumption ii) where we can observe different penetration depths for each PTL structure, which depend on the radius, the porosity, and the respective pore density. However, at high current density, we can observe that the penetration depth for some PTL/current combinations (especially the high porosity sample 200 µm 43%) reaches negative values, indicating that this assumption is not correct.

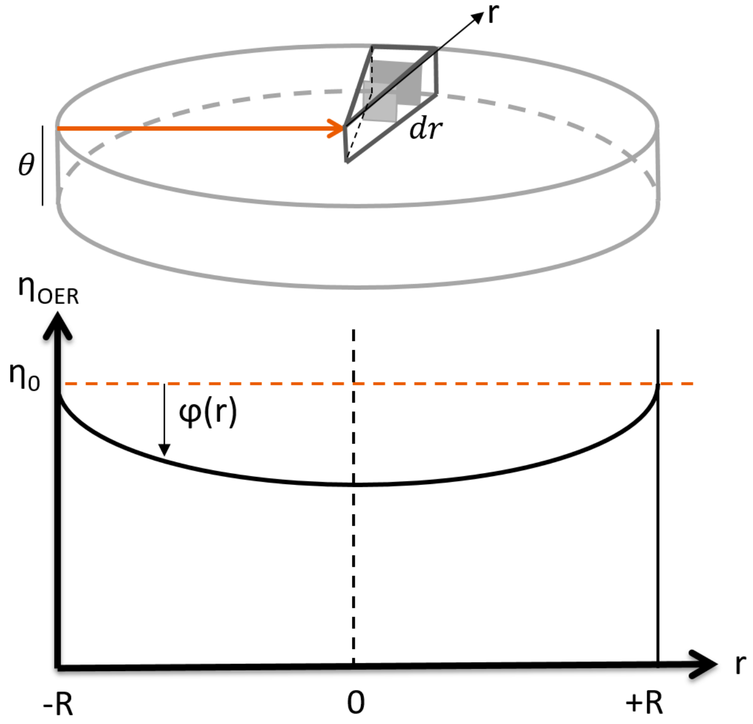

Figure S6. Schematic illustration of how the activity in the catalyst layer at the open pore space is calculated based on equations 6 & 7 in the main manuscript.

Figure S6 shows a schematic illustration of how the activity in the open pore space is calculated in Figure 5 of the main manuscript. The poor electronic conductivity of the CL affects the electrochemical reaction by a gradual potential drop across the open pore:

$$\eta_{OER}(r) = \eta_0 - \varphi(r) \quad (2)$$

This potential drop $\varphi(r)$ is effectively a nonlinear reduction of the overpotential along the cross-section of the pore (which is displayed in the lower graph of Figure S6). η_0 is the overpotential at the edge of the pore and $\eta_{OER}(r)$ at the respective distance r from the pore edge. Assuming that the OER overpotential follows Tafel behavior:

$$\eta_{OER}(r) = b \log\left(\frac{i}{i_0}\right) \quad (3)$$

With i as the nominal current density and i_0 as the exchange current density (0.6 x 10^{-9} A/cm_{Ir}^2)[2] and b the Tafel slope (which can be found in table 2 of the main manuscript). We can then calculate the radially varying electronic potential $\varphi(r)$ by solving the following ordinary differential equation:

$$\frac{d^2}{dr^2}\varphi(r) = \frac{i_0}{\theta * \sigma_e} * 10^{\frac{\eta_0 - \varphi(r)}{b}} \quad (4)$$

Where θ is the CL thickness (~10 μm), σ_e the CL electrical IP conductivity (~ 0.1 S/cm)[3]. The results for the three different pore sizes are displayed in Figure 5 of the main manuscript.

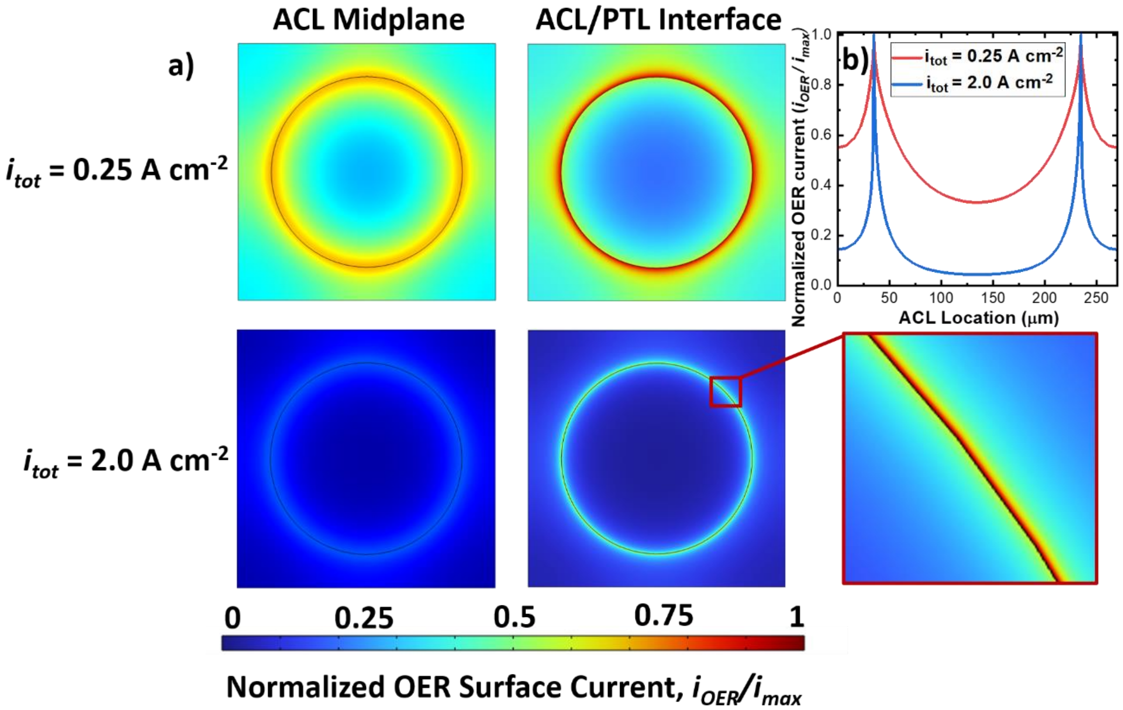

Figure S7. a) Normalized OER surface current at low (0.25 A/cm^2) and high (2.0 A/cm^2) total current densities and at two different through-plane locations of the anodic catalyst layer (aCL): the midplane of the ACL (left) and at the aCL/2D-PTL interface (right). b) 1D cross-section of the normalized OER surface current at the aCL/PTL interface.

In Figure S7 a) the normalized OER surface current for two different total current densities (low 0.25 A/cm^2 and high 2.0 A/cm^2) and at two different anodic catalyst layer (ACL) through plane locations are displayed. The normalized OER surface current represents the OER surface current density normalized to the maximum surface current density. The maximum surface current density corresponds to the peaks of the 1D cross-sections in Figure 6b) of the main manuscript. On the left side, we see the calculation for the aCL midplane (~5 µm from the interfaces), and on the right side at the anodic CL/PTL interface. In Figure S7 b) the respective 1D cross-sections of the normalized OER surface current are shown. Similar to Figure 6 of the manuscript, we can observe that at high current density, the surface current density distribution is much more inhomogeneous and more concentrated at the edge of the pore than at lower current density. This is more pronounced at the ACL/PTL interface than at the ACL midplane location.

References

(1) Kang, Z.; Mo, J.; Yang, G.; Retterer, S. T.; Cullen, D. A.; Toops, T. J.; Green Jr, J. B.; Mench, M. M.; Zhang, F.-Y. Investigation of Thin/Well-Tunable Liquid/Gas Diffusion Layers Exhibiting Superior Multifunctional Performance in Low-Temperature Electrolytic Water Splitting. *Energy Environ. Sci.* **2017**, *10* (1), 166–175. https://doi.org/10.1039/C6EE02368A.

(2) Schuler, T.; Kimura, T.; Schmidt, T. J.; Büchi, F. N. Towards a Generic Understanding of Oxygen Evolution Reaction Kinetics in Polymer Electrolyte Water Electrolysis. *Energy Environ. Sci.* **2020**, 10.1039.D0EE00673D. https://doi.org/10.1039/D0EE00673D.

(3) Schuler, T.; Ciccone, J. M.; Krentscher, B.; Marone, F.; Peter, C.; Schmidt, T. J.; Büchi, F. N. Hierarchically Structured Porous Transport Layers for Polymer Electrolyte Water Electrolysis. *Adv. Energy Mater.* **2020**, *10* (2), 1903216. https://doi.org/10.1002/aenm.201903216.
